# Tailored Supramolecular Cage for Efficient Bio-Labeling

**DOI:** 10.3390/ijms24032147

**Published:** 2023-01-21

**Authors:** Dongdong An, Linlin Shi, Tianyu Li, Hong-Yu Zhang, Yahong Chen, Xin-Qi Hao, Mao-Ping Song

**Affiliations:** 1 College of Chemistry, Zhengzhou University, Zhengzhou 450001, China; 2 College of Chemistry and Chemical Engineering, Zhoukou Normal University, Zhoukou 466001, China; 3 School of Basic Medical Science, Zhengzhou University, Zhengzhou 450001, China

**Keywords:** supramolecular cages, lanthanides, biotoxicity, bioimaging

## Abstract

Fluorescent chemosensors are powerful imaging tools used in a broad range of biomedical fields. However, the application of fluorescent dyes in bioimaging still remains challenging, with small Stokes shifts, interfering signals, background noise, and self-quenching on current microscope configurations. In this work, we reported a supramolecular cage (**C_A_**) by coordination-driven self-assembly of benzothiadiazole derivatives and Eu(OTf)_3_. The **C_A_** exhibited high fluorescence with a quantum yield (QY) of 38.57%, good photoluminescence (PL) stability, and a large Stokes shift (153 nm). Furthermore, the CCK-8 assay against U87 glioblastoma cells verified the low cytotoxicity of **C_A_**. We revealed that the designed probes could be used as U87 cells targeting bioimaging.

## 1. Introduction

Self-assembly allows for the efficient construction of supramolecular structures from relatively simple components. As a class of such architectures, metal-organic cages (MOCs) have gained significant attention in recent years [1,2,3,4]. Numerous MOCs exhibit astonishing multifunctional capabilities that have been created by regulating and controlling the coordination environment surrounding the core metal ions, as well as the suitable choice of organic ligands. Consequently, they have found applications in areas such as catalysis [5,6,7,8,9,10], molecular recognition [11,12,13,14,15,16,17], biological imitation [18,19,20,21,22], guest sequestration [23,24,25], reactive species stabilization [26], drug delivery [27,28,29], and membrane transportation [30,31], among others.

The diagnosis and treatment of disease require better and faster pathological analysis, as well as highly contrasting real-time biological images. Optical emission probes combined with disease diagnosis and treatment are becoming strong candidates, owing to their only slight disturbance of the systems and organs being researched. When the appropriate wavelength is used, the penetration depth of optical emission probes can be substantial, and then the light can reach regions of complex molecular structures that are inaccessible to other molecular probes. As a consequence, building supramolecular structures, which have strong fluorescence to achieve deeper tissue penetration for imaging or cell tracking studies to monitor biological processes, has recently attracted increasing attention [32,33]. Significantly, lanthanide ions with special optical and magnetic properties serve as functional centers on which to build the supramolecular structures, and which have found uses across various fields [34,35], such as luminous probes [36,37,38,39], analyte sensing [40,41], and tissue [42,43]. Furthermore, the pharmaceutical properties of lanthanide (III) ionic have also been demonstrated as antibacterial agents (anticancer, anticoagulant, anti-inflammatory, etc.), and play a crucial role in medicine, especially cancer diagnosis and treatment [44]. However, the difficulty of predicting the coordination numbers of lanthanides and coordination geometry makes the controlled self-assembly of predetermined structures quite challenging. Most studies have been limited to small systems, such as single-core beams or two-core helixes; the design and the application of three-dimensional lanthanide components have not been thoroughly studied.

**Figure 1:**
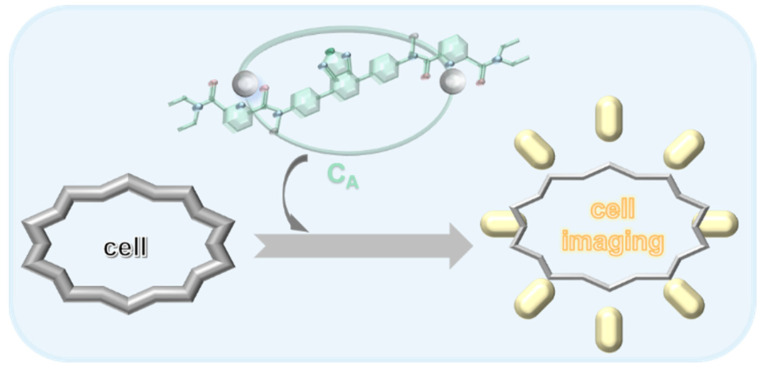
The preparation and characterization of **C_A._**

In this context, we report the synthesis of three-dimensional (3D) dimetallic lanthanide complexes with saturated coordination spheres. Two-armed tri-toothed benzothiadiazole derivatives acted as organic ligands and coordination-driven self-assembly with Eu(OTf)_3_ in a ratio of 2:3 to form a single structure. Trivalent lanthanides display a tendency to adopt nine-coordinate tricapped trigonal prismatic (TTP) arrangements around the metal ion in the solid state, and the 3D molecular buildings are capable of great fluorescence. Well-defined solid clusters enable this supramolecular cage structure to efficiently target the staining of living cancer cells with low biotoxicity (Figure 1).

## 2. Results and Discussion 

### 2.1. The Preparation and Characterization of **C_A_**

**Scheme 1:**
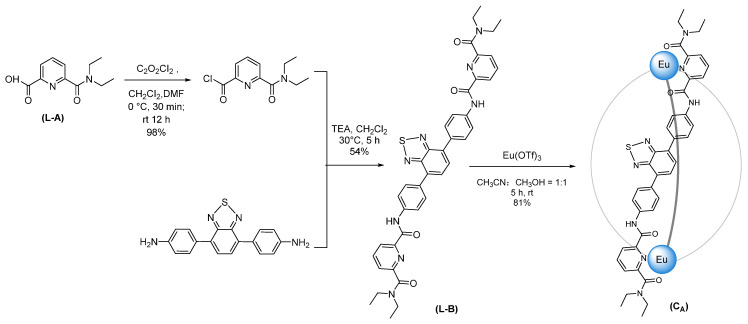
Synthesis of ligand (**L-B**) and self-assembly of metal-organic cage (**C_A_**).

Highly predisposed O-N-O tridentate chelators with amide groups were introduced into linear shape molecules *N^2^,N^2’^*-(benzo[c][1,2,5]thiadiazole-4,7-diylbis(4,1-phenylene))bis(*N^6^,N^6^*-diethylpyridine-2, 6-dicarboxamide) (**L-B**), to control the coordination of lanthanide ions and obtain highly ordered architectures [45,46,47]. The **L-B** was synthesized by amide condensation of freshly prepared 6-(diethylcarbamoyl)picolinic acid with 4, 4’-(benzo[c][1,2,5]thiadiazole-4, 7-diyl)dianiline. The formation of the supramolecular cage was quantitatively carried out, **L-B** (3 equiv.) and Eu(OTf)_3_ (2 equiv.) were dissolved in 600 μL CH_3_CN/CH_3_OH (*v*/*v*, 1/1), respectively, and stirred in a 10 mL flask at room temperature for 5 h. After the reaction, 5 mL diethyl ether was added and centrifuged to obtain **C_A_** as an orange-red solid (Scheme 1 and Figures S1–S9).

**Figure 2:**
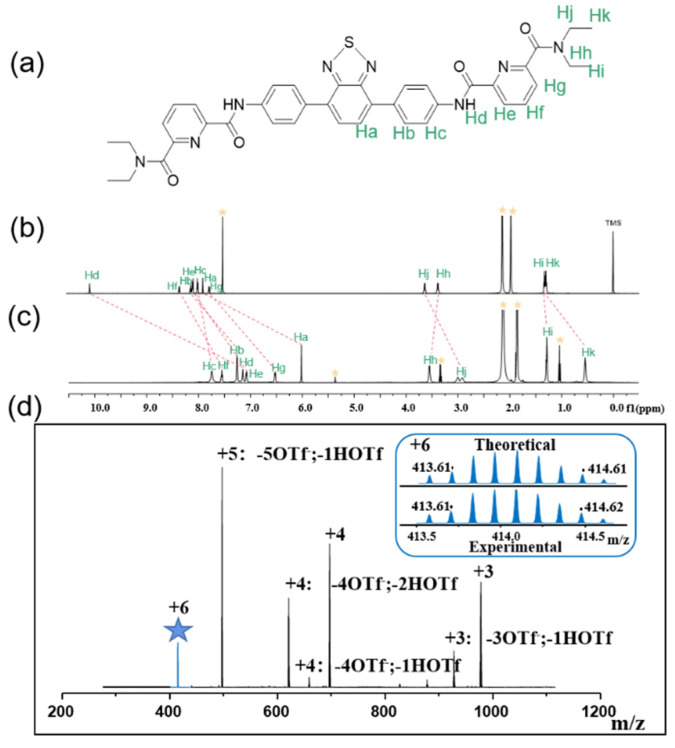
(**a**) The structure of **L-B**. (**b**) ^1^H NMR spectra (600 MHz, CD_3_CN/CDCl_3_ = 1:1), 298 K) of **L-B** (the solubility of **L-B** in CD_3_CN was poor, and CDCl_3_ was added to help dissolve). (**c**) ^1^H NMR spectra (600 MHz, CD_3_CN, 298 K) of **C_A_** [the yellow five-pointed star label indicates the solvent peak]. (**d**) ESI-MS spectra of **C_A_** and showing the observed z = +6 charge for the peak at *m/z* = 414.11 (bottom) compared to the theoretical isotopic pattern (top).

Multinuclear NMR (^1^H, DOSY, COSY, and NOSEY) analysis (Figure 2 and Figures S10–S14), IR spectrum, and electrospray ionization time-of-flight mass spectrometry (ESI-TOF-MS) confirmed the formation of **C_A_**. The ^1^H NMR spectrum of **C_A_** displayed only one set of ligand signals (Figure 2c), showing the formation of faces and vertices with a single stereochemical orientation. The shift in NMR signal could be observed after the formation of the supramolecular cage. As evidenced by the results, upfield chemical shifts were observed for the β-pyridyl protons He (from 8.066 to 7.146 ppm) and Hg (from 7.74 to 6.61 ppm) and γ-pyridyl protons Hf (from 8.324 to 7.629 ppm). Chemical displacement changes could also be observed in amide hydrogen Hd (from 10.052 to 7.225 ppm). Moreover, aromatic protons Ha, Hb, and Hc, belonging to 4,4’-(benzo[c][1,2,5]thiadiazole-4,7-diyl)dianiline, shifted upfield as well (Figure 2b,c). All of these chemical shift changes pointed to the formation of **C_A_**. 2D DOSY NMR also revealed the formation of a single species in solution, with a diffusion coefficient of 4.79 × 10^−10^ m^2^/s. The infrared spectrum also confirmed the successful coordination-driven self-assembly of **C_A_** (Figures S15 and S16). According to the results, the characteristic peaks of C=O (from 1686 to 1601 cm^−1^) and N-H (from 1625 to 1575 cm^−1^) were significantly changed.

Additionally, electrospray ionization-mass spectrometry (ESI-MS) indicated the successful assembly of **C_A,_** and the detected ESI-MS data of the resultant cage was nearly identical to the theoretical data. The spectra showed a series of peaks with charge states ranging from 3^+^ to 6^+^, resulting from the sequential loss of OTf^-^ counterions or HOTf molecules. For example, peaks with *m/z* = 414.11 correspond to [Eu_2_L_3_(OTf)_6_-6OTf^-^]^6+^ charged species. (Figure 2d). The most abundant peak at m/z = 496.84 exhibited typical m/z splitting for a 5^+^ charged species and was assigned to [Eu_2_L_3_(OTf)_6_-6OTf^-^-HOTf]^5+^. The average molar mass of the self-assembled cage **C_A_** after deconvolution was 3378 Da, which was in good agreement with their predicted chemical compositions [C_126_H_114_N_24_O_30_F_18_S_9_Eu_2_] and ruled out the formation of any subsequent undesirable complexes (Figures S17–S19).

**Figure 3:**
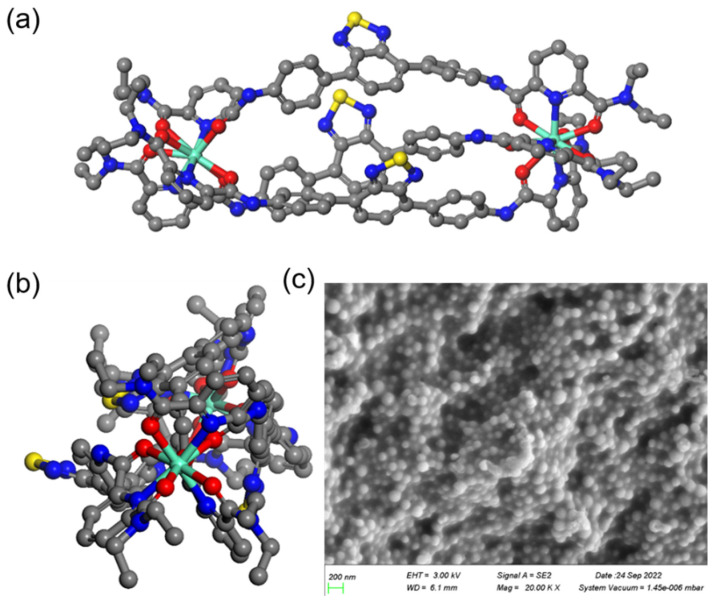
Energy-minimized molecular model of **C_A_** (Eu, green; N: blue; S, yellow; O, red; C, gray). Hydrogens, counteranions are omitted for clarity; (**a**) side view and (**b**) top view. (**c**) TEM images of **C_A_**.

The complete data for single crystal X-ray analysis could not be obtained despite numerous attempts. Consequently, we developed a computerized molecular model of the **C_A_** (Figure 3a,b). Two europium ions were nine-coordinated by tridentate O-N-O chelating moieties from three distinct ligands in the energy-minimized structure. The metal-metal distance between europium ion centers in the energy-minimized structure was 19.17 Å (as measured by Materials Studio.) Contrary to **L-B**, **C_A_** had a geometry-optimized structure that revealed one of the ligands to be severely twisted out of the ligand plane, which prevented a cavity from forming in the supramolecular cage. Scanning electron microscopy (SEM) revealed that **C_A_** was in a good state of aggregation, and showed a uniform spherical morphology (Figure 3c). This matched the molecular structure that we modeled.

### 2.2. Optical Properties

**Figure 4:**
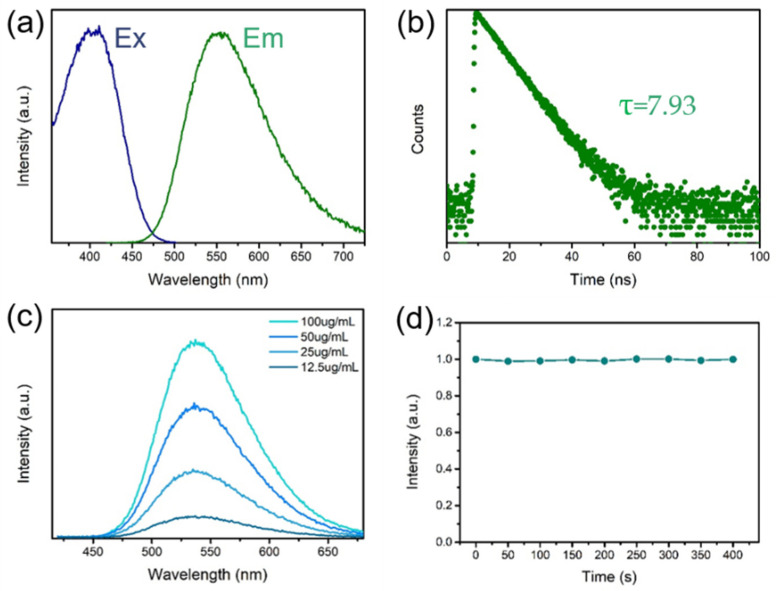
Optical properties of **C_A_** in acetonitrile. (**a**) PLE and PL emission spectra of **C_A_** (c = 50 μg/mL). (**b**) Time-resolved decay spectra of **C_A_** (c = 50 μg/mL). The data were collected at an emission peak of 553 nm (λex = 400 nm). (**c**) Variable concentration PL spectra of **C_A_**. (**d**) PL photostability tests of **C_A_** (c = 50 μg/mL, λex = 400 nm λem = 537 nm). The samples were treated with the irradiation of a 400 nm blue light.

The enormous Stokes shift and the outstanding PL QY are the distinguishing characteristics of **C_A_**. Figure 4 shows the optical properties of **C_A_** in acetonitrile. The UV-Vis absorption spectra (Figure S20) showed that the **C_A_** possessed continuous absorption from the UV region to about 475 nm. The absorption bands at 210–250 nm could be ascribed to π-π* transition from aromatic C=C bonds; a peak at 275–340 nm was also observed, which may be attributed to the *n*-π* transition of C=O on the surface of **C_A_**. Absorbance significantly increased compared with **L-B**, and an M-shaped peak appeared, which was attributed to the interaction between C=O bonds and Eu (III) ions within the process of self-assembly. The absorption peak located at 375–450 nm was assigned to the C=N absorption of pyridine-N in the carbon core and the polycyclic aromatic units. The excitation spectrum of **C_A_** showed the best excitation wavelength of 400 nm, and the PL emission peak was centered at 553 nm (Figure 4a). Due to the **C_A_** having a large Stokes shift reaching 153 nm, the small self-absorption of **C_A_** could be expected, which enabled efficient PL emission. Under the optimum excitation wavelength, the absolute PL quantum yield of **C_A_** was 38.57% (Figure S21). As illustrated, the decay profiles fit single exponential functions, giving lifetimes of 7.93 ns for **C_A_** (Figure 4b). The emission spectrum of **C_A_** with different concentrations demonstrated that the change of concentration did not affect maximum emission (Figure 4c). Additionally, we explored **C_A_**’s PL stability (Figure 4d) under the influence of a 400 nm blue light for 400 s; the PL intensity of the **C_A_** solution remained constant. Therefore, the supramolecular cage structure showed surprising advantages in terms of a significant Stokes shift, an extremely high PL QY, and good fluorescence stability, all of which are advantageous for applications.

### 2.3. In Vitro Cytotoxicity Profiles and CLSM Images

**Figure 5:**
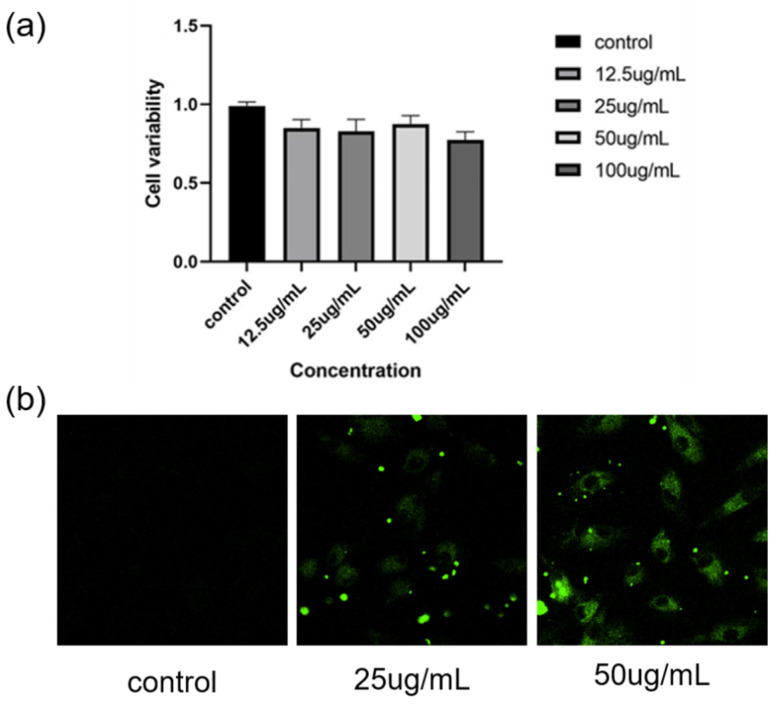
(**a**) In vitro U87 cytotoxicity profiles of **C_A_**. Mean values and error bars were defined as mean and s.d., respectively. Experiments were performed in triplicate. (**b**) CLSM images of viable cell distributions after co-incubation with **C_A_** at varied concentrations: 0, 25, and 50 μg/mL for 4 h.

After the addition of DMEM medium, it was found that the characteristic peaks of **C_A_** did not change (Figure S22). Therefore, the structure of **C_A_** remained stable during the process of biological experiments. The cytotoxic activity of the **C_A_** against U87 cells was evaluated. Cultured cancer cells were treated with concentrations of 0, 12.5, 25, 50, and 100 μg/mL of **C_A_** in basal medium. The percentage of cell viability was determined by Cell Counting Kit-8 (CCK-8) assay. In the U87 cytotoxicity assay, the **C_A_** presented 84.95, 82.97, 87.27, and 77.43% cell viability at increasing concentrations (Figure 5a), and the results showed good biosafety. Then, we used confocal laser scanning microscopy to evaluate the suitability of low-toxicity **C_A_** (25 μg/mL, and 50 μg/mL) for bioimaging. An intracellular fluorescence signal from **C_A_** was clearly observed (Figure 5b), the fluorescence intensity gradually increased in line with the increase in concentration of **C_A_**, while no emission was found in the control group. Therefore, **C_A_** with 50 μg/mL concentration was selected for cell imaging to achieve accurate labeling of U87 glioblastoma cells.

## 3. Materials and Methods

### 3.1. Materials

All reagents were purchased from Sigma-Aldrich, Shanghai, China, Fisher, Shanghai, China, Acros, Shanghai, China, Bide Pharmatech Ltd., Shanghai, China, and, Alfa Aesar, Tianjin, China, and were used without further purification. All solvents were dried according to standard procedures and all of them were degassed under N_2_ for 30 min before use.

### 3.2. Measurements

^1^H NMR and ^13^C NMR spectra were measured on a Bruker AV 600 spectrometer. ESI-MS was recorded with a Waters Synapt G2-Si mass spectrometer, USA, in ESI mode. The UV–Vis spectra were recorded on a dual-beam UV–Vis spectrophotometer (TU-1901), PERSEE, Beijing, China. Fluorescent spectra were collected on a HORIBA FluoroLog-3 fluorescence spectrometer and Horiba-FluoroMax-4 spectrofluorometer, HORIBA, Edison, NJ, USA. The luminescence lifetimes were measured on an Edinburgh FLS 980 fluorescence spectrometer, Edinburgh Instruments, UK. The photoluminescence quantum yield (PLQY) was measured using an integrating sphere on a HORIBA FluoroLog-3 fluorescence spectrometer, HORIBA, Edison, NJ, USA. The Fourier Transform Infrared FT-IR spectra were recorded on a Spectrum TWO FT-IR spectrophotometer, PerkinElmer, Llantrisant, UK.

### 3.3. Materials Synthesis

#### 3.3.1. Preparation of 2-Ethoxycarbonyl-carboxypyridine

Dipicolinic acid (5.00 g, 32.9 mmol) was refluxed for 4 h in methanol: water mixture (50 mL/50 mL) containing concentrated H_2_SO_4_ (5 mL). The cooled solution was poured onto saturated NaHCO_3_ (500 mL) and the aq. phase was extracted with dichloromethane (4 × 100 mL). Acidification of the resulting aq. phase (pH = 2) with concentrated hydrochloric acid (pH = 2) followed by extraction with dichloromethane (4 × 100 mL) provided a second organic phase, which was dried (anhydrous Na_2_SO_4_) and evaporated to dryness to afford 2-ethoxycarbonyl-carboxypyridine (3.51 g, 59% 19.4 mmol) as a white powder. ^1^H NMR (600 MHz, CD_3_OD) δ 8.33 (dd, *J* = 18.5, 7.5 Hz, 2H), 8.17 (t, *J* = 7.8 Hz, 1H), 4.02 (s, 3H). ^13^C NMR (151 MHz, CDCl_3_) δ 164.2, 163.5, 146.8, 146.5, 139.6, 128.8, 126.9, 53.2.

#### 3.3.2. Preparation of **L-A**

Oxalyl chloride (1.68 g, 13.25 mmol) and 2-ethoxycarbonyl-carboxypyridine (2.00 g, 11.04 mmol) were dissolved in DMF (0.1 mL) and dry dichloromethane (100 mL) at 0 °C for 30 min; the whole reaction mixture was stirred at room temperature for 12 h. The resulting mixture was evaporated to dryness, and dissolved in dry dichloromethane (50 mL), then, *N*, *N*-diethylamine (17.10 mL, 16.50 mmol), and stoichiometric excess Et_3_N were added dropwise at room temperature and the solution was stirred for 5 h. The solution was washed with portions of 1 N HCl, followed by 1 N NaOH, and, finally, water. The organic layer was isolated, dried over MgSO_4_, and concentrated by rotary evaporation. Diethyl ether was added to precipitate the product as white precipitates, which were collected by filtration and dried. The compound obtained above was then dissolved into a solution of 1 N KOH and stirred for 1 h at room temperature. After washing with dichloromethane (2 × 50 mL), the aq. phase was neutralized (pH = 2) with concentrated hydrochloric acid and kept at 4 °C for 12 h. The mixture was extracted with dichloromethane (3 × 50 mL), dried over anhydrous Na_2_SO_4_, filtered, and then concentrated under reduced pressure to afford 6-(*N*,*N*-diethylcarbamoyl)pyridine-2-carboxylic acid (**L-A**, 1.69 g, 69% 7.62 mmol) as a white powder. ^1^H NMR (600 MHz, CD_3_OD) *δ* 8.21 (d, *J* = 7.8 Hz, 1H), 8.10 (t, *J* = 7.8 Hz, 1H), 7.76 (d, *J* = 7.7 Hz, 1H), 3.58 (q, *J* = 7.1 Hz, 2H), 3.33 (q, *J* = 7.1 Hz, 2H), 1.28 (t, *J* = 7.1 Hz, 3H), 1.19 (t, *J* = 7.1 Hz, 3H). ^13^C NMR (151 MHz, CDCl_3_) *δ* 166.8, 163.6, 154.0, 145.0, 140.0, 127.2, 124.4, 43.2, 40.3, 14.5, 12.8.

#### 3.3.3. Preparation of **L-B**

**L-A** (111.05 mg, 0.50 mmol) and Oxalyl chloride (76.15 mg, 0.60 mmol) were dissolved in DMF (0.1 mL) and dry dichloromethane (30 mL) at 0 °C for 30 min; the whole reaction mixture was stirred at room temperature for 12 h, and then concentrated by rotary evaporation. Then, 4,4’-(benzo[1,2,5]thiadiazole-4,7-diyl)dianiline (53.10 mg, 0.17 mmol) was added dropwise at room temperature and the solution was stirred for 5 h. The solution was washed with portions of 1 N HCl, followed by 1 N NaOH and, finally, saturated sodium chloride solution. The organic layer was isolated, dried over MgSO_4_, and concentrated to 5 mL by rotary evaporation. The mixture was poured into diethyl ether (50 mL) to give a precipitate, which was collected by centrifugation to afford **L-B** (65.40 mg, 54% 0.09 mmol) as a yellow powder. ^1^H NMR (600 MHz, CDCl_3_/CD_3_CN = 1:1) *δ* 10.05 (s, 1H), 8.33 (d, *J* = 7.6 Hz, 1H), 8.11 (t, *J* = 7.7 Hz, 1H), 8.07 (d, *J* = 8.6 Hz, 2H), 7.98 (d, *J* = 8.6 Hz, 2H), 7.88 (s, 1H), 7.76 (d, *J* = 7.7 Hz, 1H), 3.62 (q, *J* = 7.0 Hz, 3H), 3.37 (q, *J* = 6.9 Hz, 2H), 1.48–1.12 (m, 7H). ^13^C NMR (151 MHz, CDCl_3_) *δ* 167.6, 161.5, 154.1, 153.7, 148.5, 138.9, 137.7, 133.6, 132.5, 130.0, 127.8, 126.0, 123.1, 119.8, 43.2, 40.2, 14.7, 12.8.

#### 3.3.4. Preparation of **C_A_**

**L-B** (13.10 mg, 0.018 mmol) and Eu(OTf)_3_ (7.19 mg, 0.012 mmol) were added to a solution of methanol: acetonitrile mixture (600 μL/600 μL); the solution was stirred at room temperature for 5 h. The reaction mixture was poured into diethyl ether (10 mL) to give a precipitate, which was collected by centrifugation to afford **C_A_** (16.40 mg, 81% 0.0048 mmol) as an orange-red solid. ^1^H NMR (600 MHz, CD_3_CN) *δ* 7.86 (s, 2H), 7.66 (t, *J* = 7.7 Hz, 1H), 7.37 (d, *J* = 6.6 Hz, 2H), 7.26 (s, 1H), 7.18 (d, *J* = 7.7 Hz, 1H), 6.64 (d, *J* = 7.9 Hz, 1H), 6.13 (s, 1H), 3.66 (d, *J* = 6.7 Hz, 2H), 3.07 (d, *J* = 51.0 Hz, 2H), 1.40 (t, *J* = 6.7 Hz, 3H), 0.66 (s, 3H).

### 3.4. Fluorescence Lifetime

The fluorescence lifetime is measured by the single exponential fitting method; the formula is as follows:

(1) y=A+B1exp(−t/t1)

### 3.5. Fluorescence Quantum Yield

The absolute fluorescence quantum yield is measured using the four-curve method; the formula is as follows:

(2) QY={Ea−[Ec(Lc/La)]}/(Lc−La)

*Ea* is sample emission area, *Ec* is blank emission area, *La* is sample excitation area, *Lc* is blank excitation area. (Scatter Range: 395.00 to 415.00 nm. Emission Range: 465.00 to 749.00 nm).

### 3.6. Cell Variability Assay

U87 glioblastoma cells were purchased from Stem Cell Bank, Chinese Academy of Sciences. The cell line was authenticated and tested free of mycoplasma. The U87 cells were grown in DMEM containing 10% fetal bovine serum and 1% penicillin-streptomycin. The cells were maintained at 37 °C in a humidified atmosphere with 5% CO_2_. For this, 5000 cells in 100 µL per well were seeded in a 96-well plate and incubated for 24 h, then the cells were treated with different concentrations of **C_A_** in basal medium, in triplicate. Following 24-h incubation, the cells were incubated with 10% CCK-8 containing DMEM high-glucose medium at 100 µL per well at 37 °C in a humidified atmosphere with 5% CO_2_. CCK-8 could selectively stain the viable cells in 1 h, read by a spectrometer at 450 nm.

### 3.7. Live Cell Imaging

U87 cancer cells were plated on 35 mm plates (1 × 10^5^ cells in 1 mL media per plate) and maintained at 37 °C in a humidified atmosphere with 5% CO_2_ for 24 h. Subsequently, the medium was discarded and the disks were rinsed with PBS twice, before 1 mL of DMEM high-glucose containing 25 μg/mL and 50 μg/mL of **C_A_** were replaced. Following 4-h incubation, forming fluorescent of **C_A_** (λex = 405 nm, λem = 546 nm) could be observed by confocal laser scanning microscopy (LSM 980 ZEISS).

## 4. Conclusions

In conclusion, we designed and synthesized a coordination-driven self-assembly supramolecular for cell imaging of live U87 glioblastoma cells. This supramolecular cage structure showed surprising advantages in terms of its unique low biotoxicity, high targeting, a large Stokes shift, high fluorescence efficiency, and strong photostability. This study will provide some guidance for the design of abundant organelle targeting probes and provide new ideas for further cancer diagnosis and treatment.

## Data Availability

Not applicable.

## Supplementary Materials

The supporting information can be downloaded at: https://www.mdpi.com/article/10.3390/ijms24032147/s1.
