# Supplementary material for: Tailored Supramolecular Cage for Efficient Bio-Labeling
Source: Int J Mol Sci. 2023 Jan 21;24(3):2147. doi: 10.3390/ijms24032147 (PMC9916613; doi:10.3390/ijms24032147)
Supplement: Supplementary file 1 [file ijms-24-02147-s001.zip › ijms-2032555-supplementary.pdf]

Supporting Information

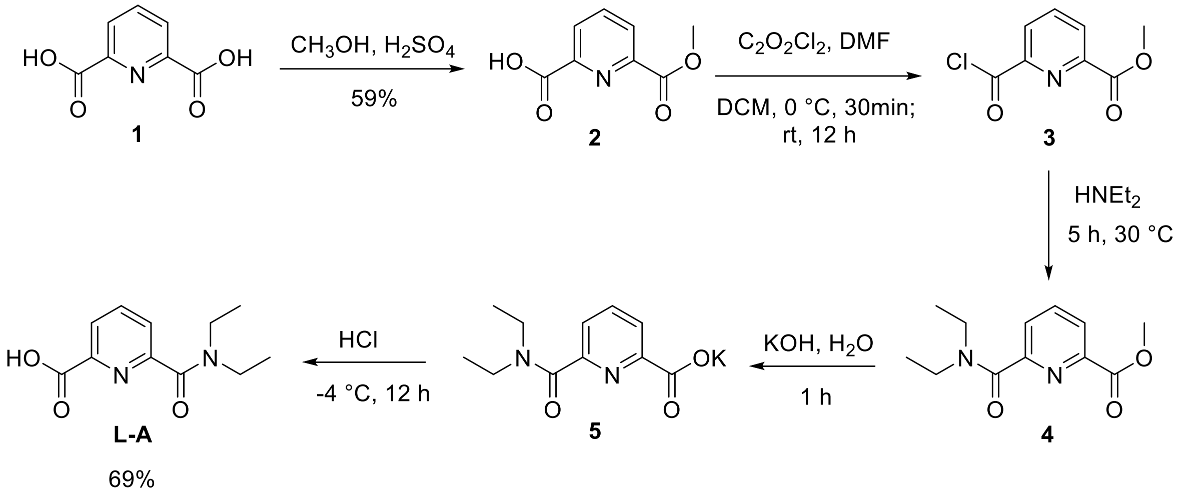

Figure S1. Synthesis of **L-A**.

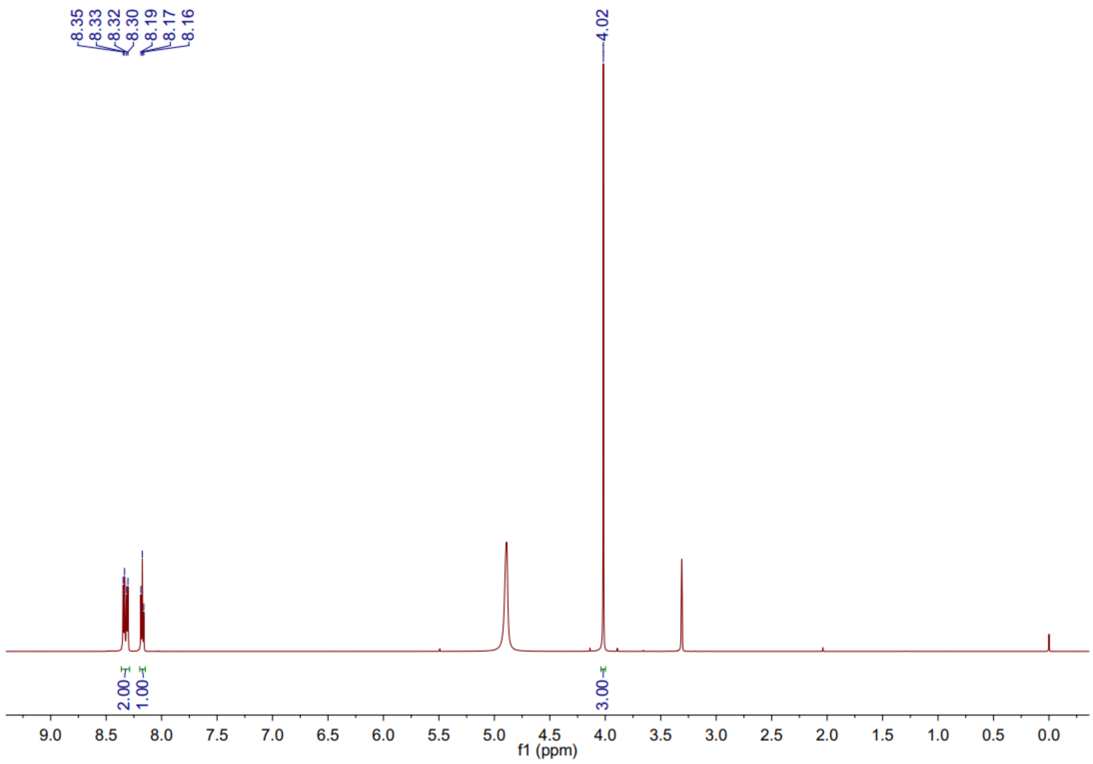

Figure S2. ^{1}H NMR (600 MHz, 298 K, CD_3OD) spectrum of **2**.

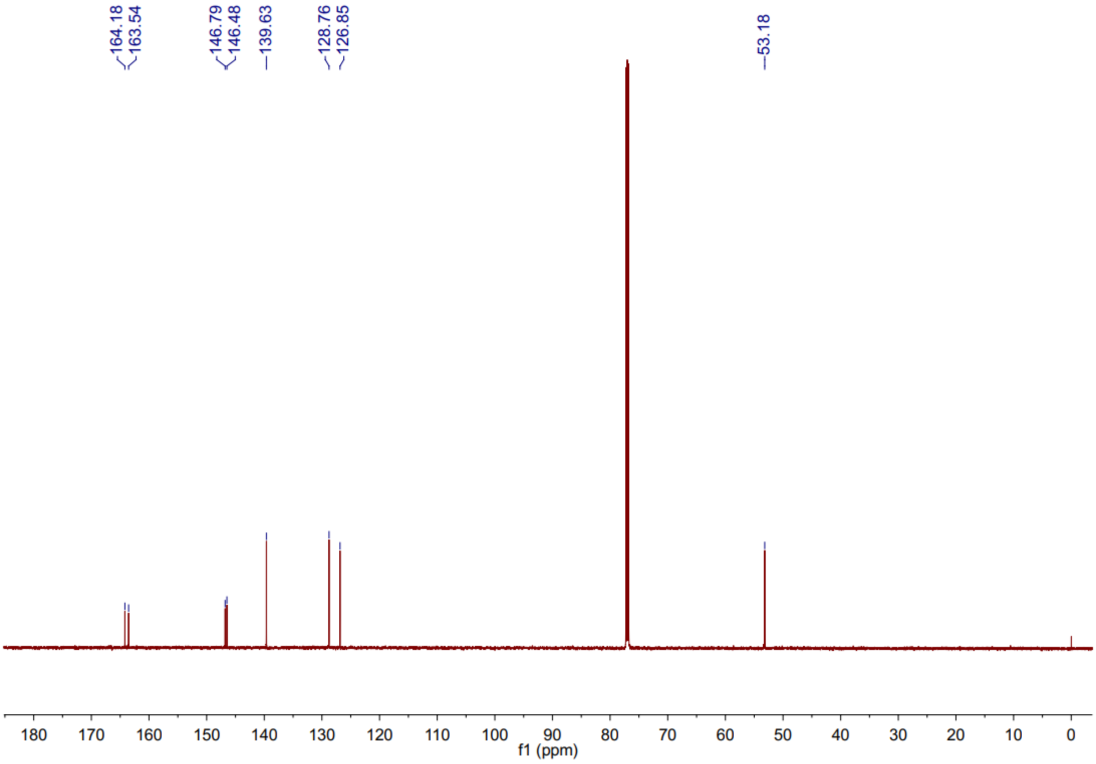

Figure S3. ^{13}C NMR (151 MHz, 298 K, CD_3OD) spectrum of **2**.

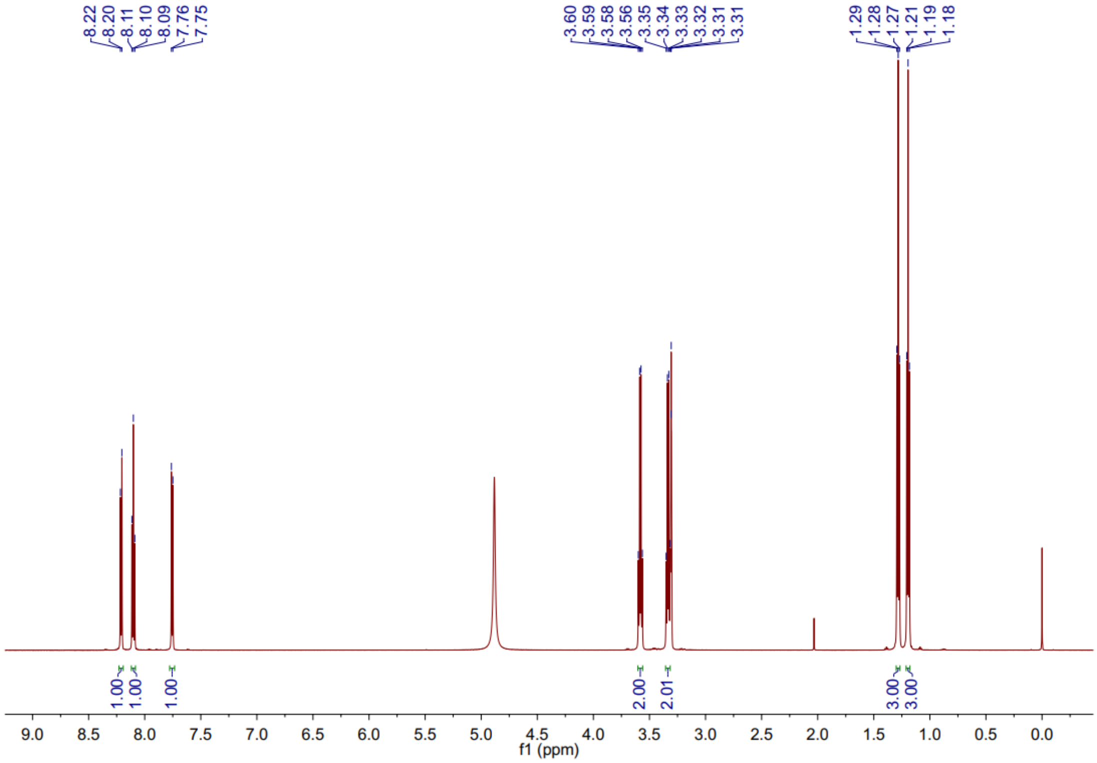

Figure S4. ^{1}H NMR (600 MHz, 298 K, CD_3OD) spectrum of **L-A**.

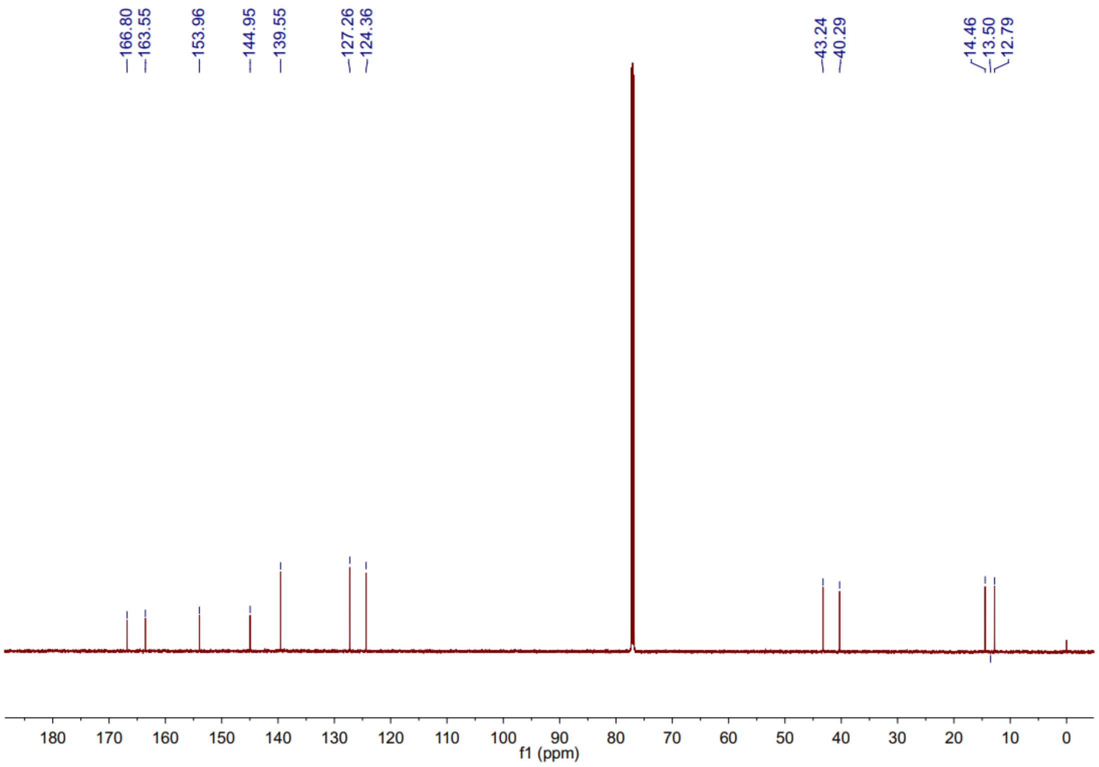

Figure S5. ^{13}C NMR (151 MHz, 298 K, $CDCl_3$) spectrum of **L-A**.

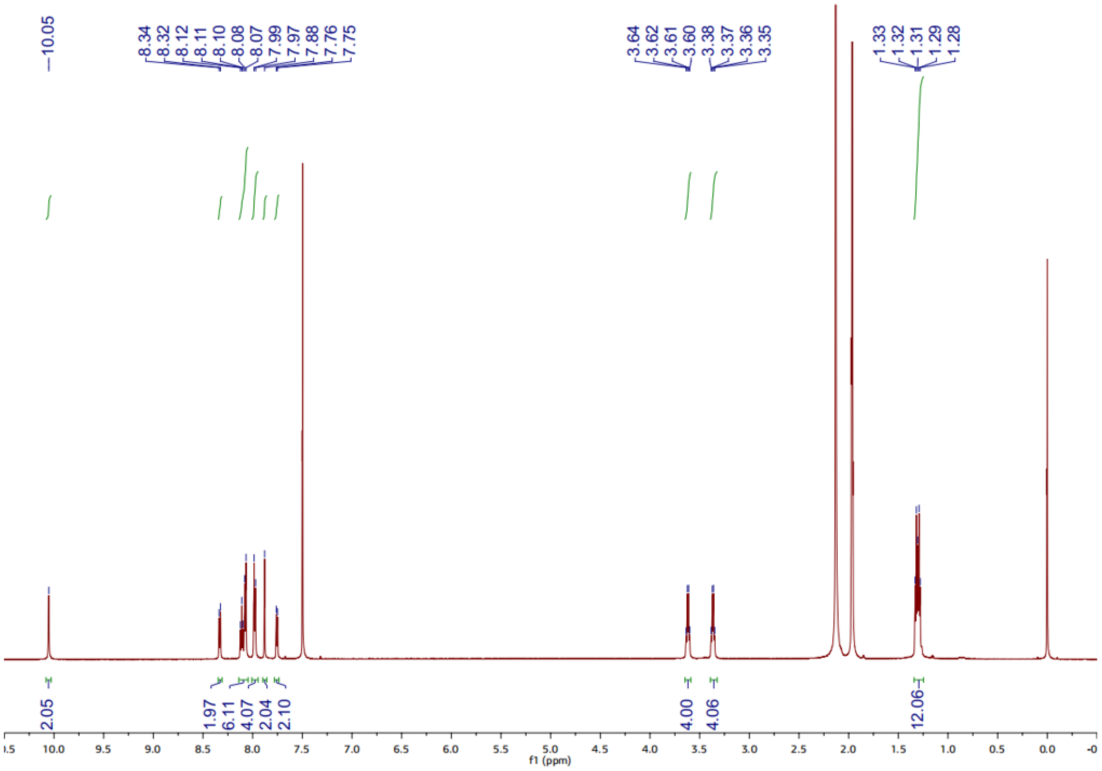

Figure S6. ^{1}H NMR (600 MHz, 298 K, $CD_3CN/CDCl_3$ = 1/1) spectrum of **L-B**.

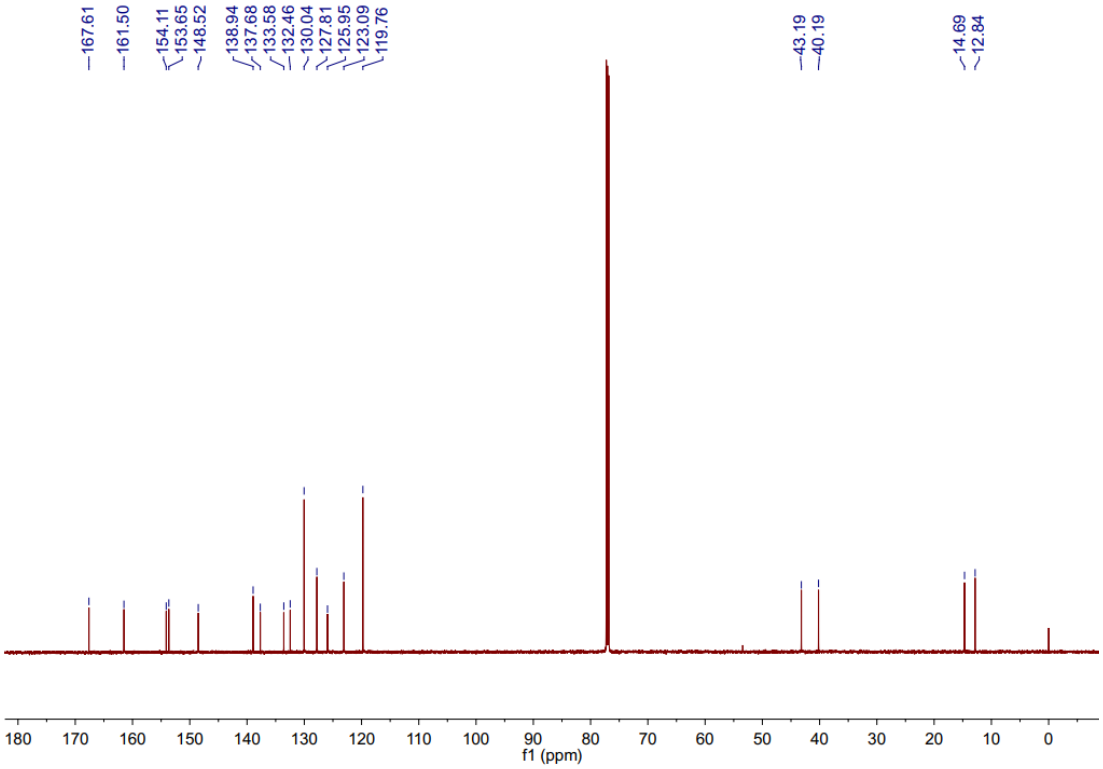

Figure S7. ^{13}C NMR (151 MHz, 298 K, $CDCl_3$) spectrum of **L-B**.

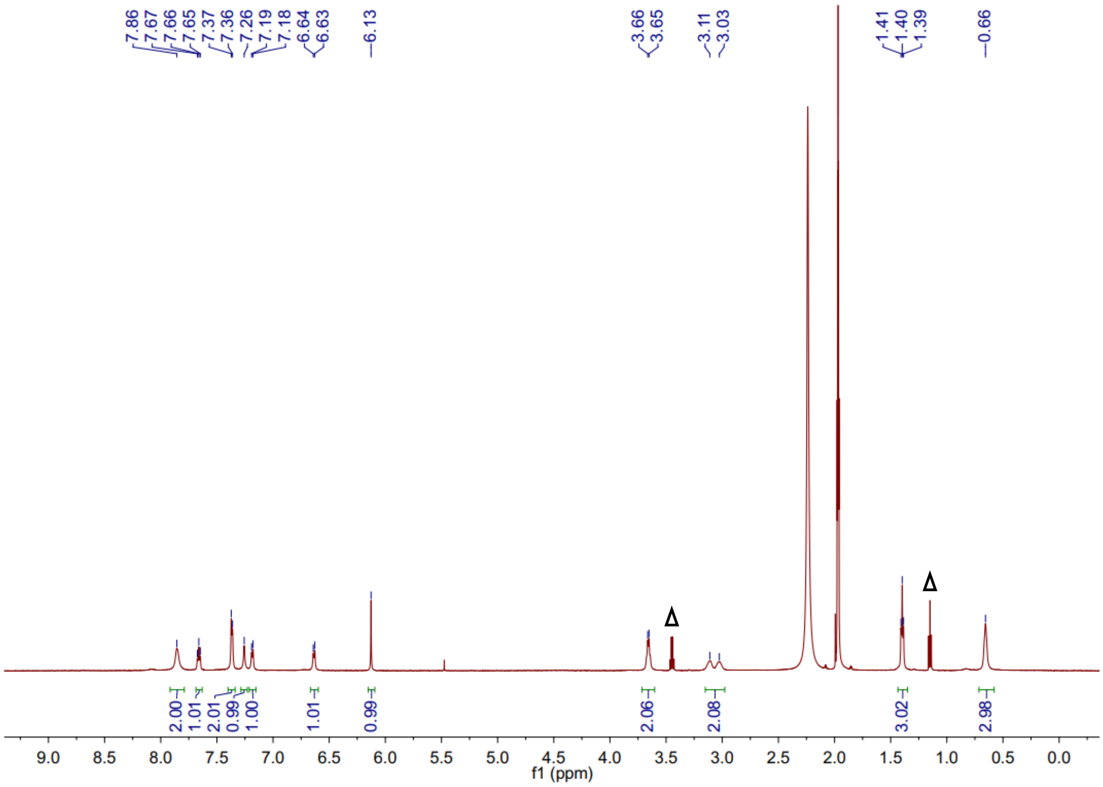

Figure S8. ^{1}H NMR (600 MHz, 298 K, CD_3CN) spectrum of **C**$_A$. (Δ=Et_2O in CD_3CN)

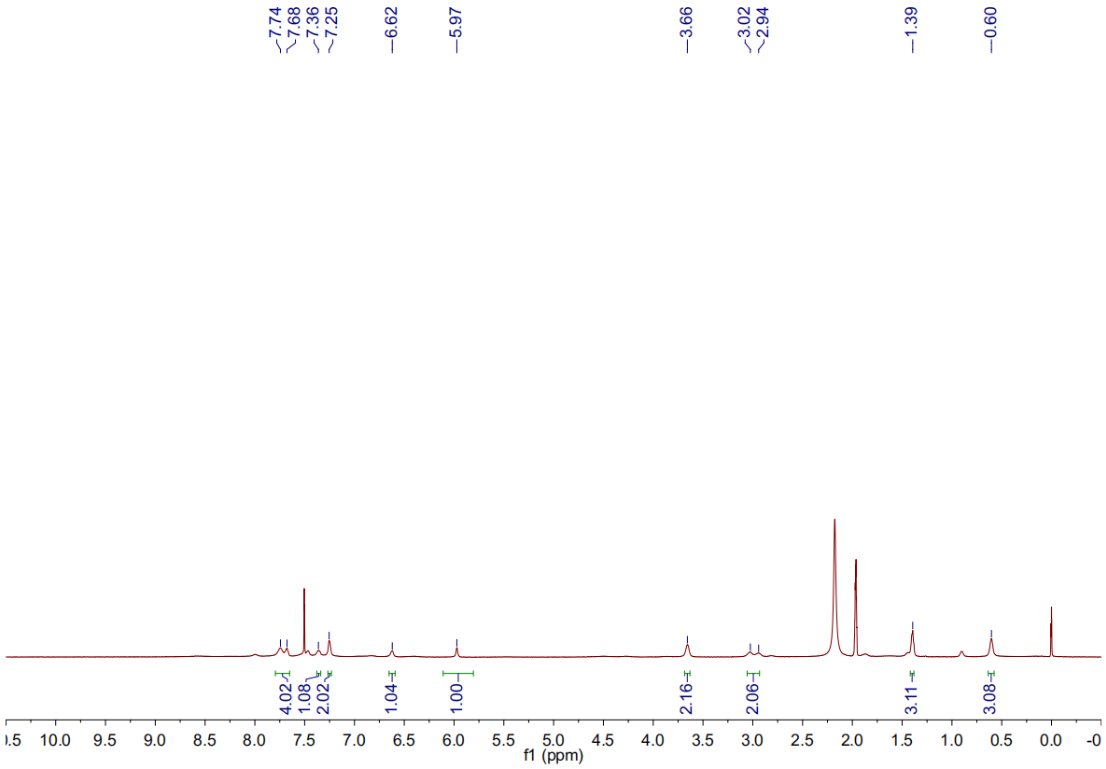

Figure S9. ^{1}H NMR (600 MHz, 298 K, $CD_3CN/CDCl_3$ = 1/1) spectrum of **CA**.

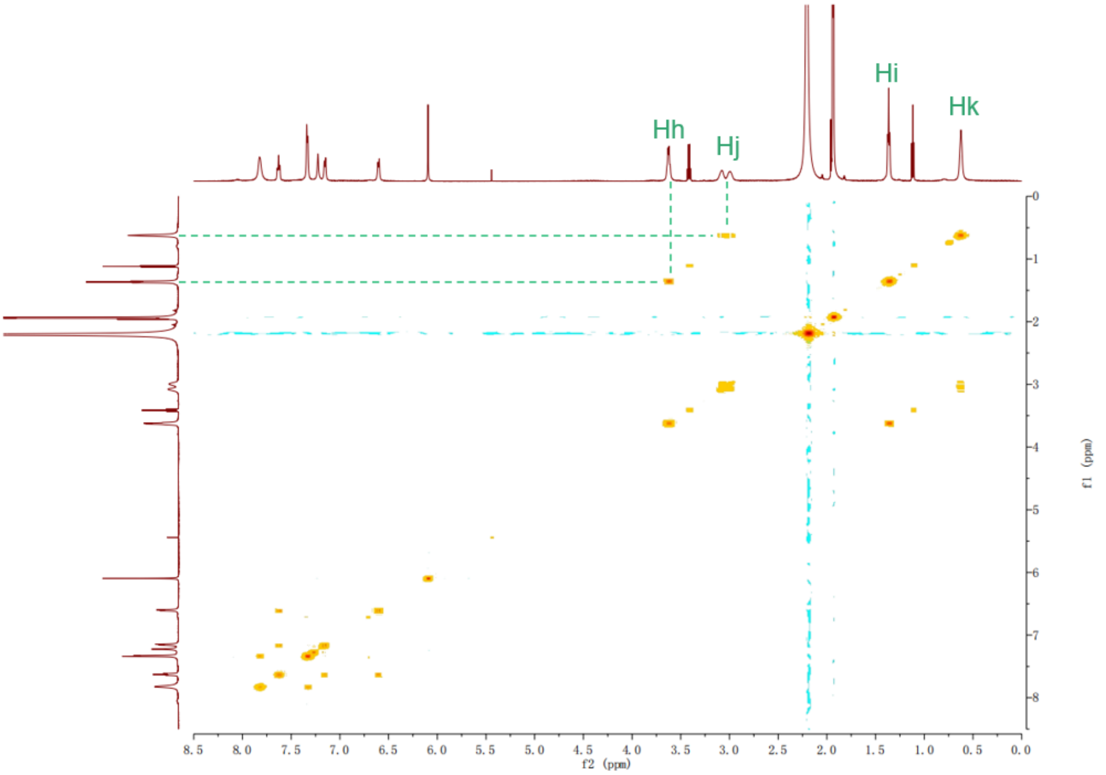

Figure S10. Aromatic region of the COSY (CD_3CN) spectrum of **CA**.

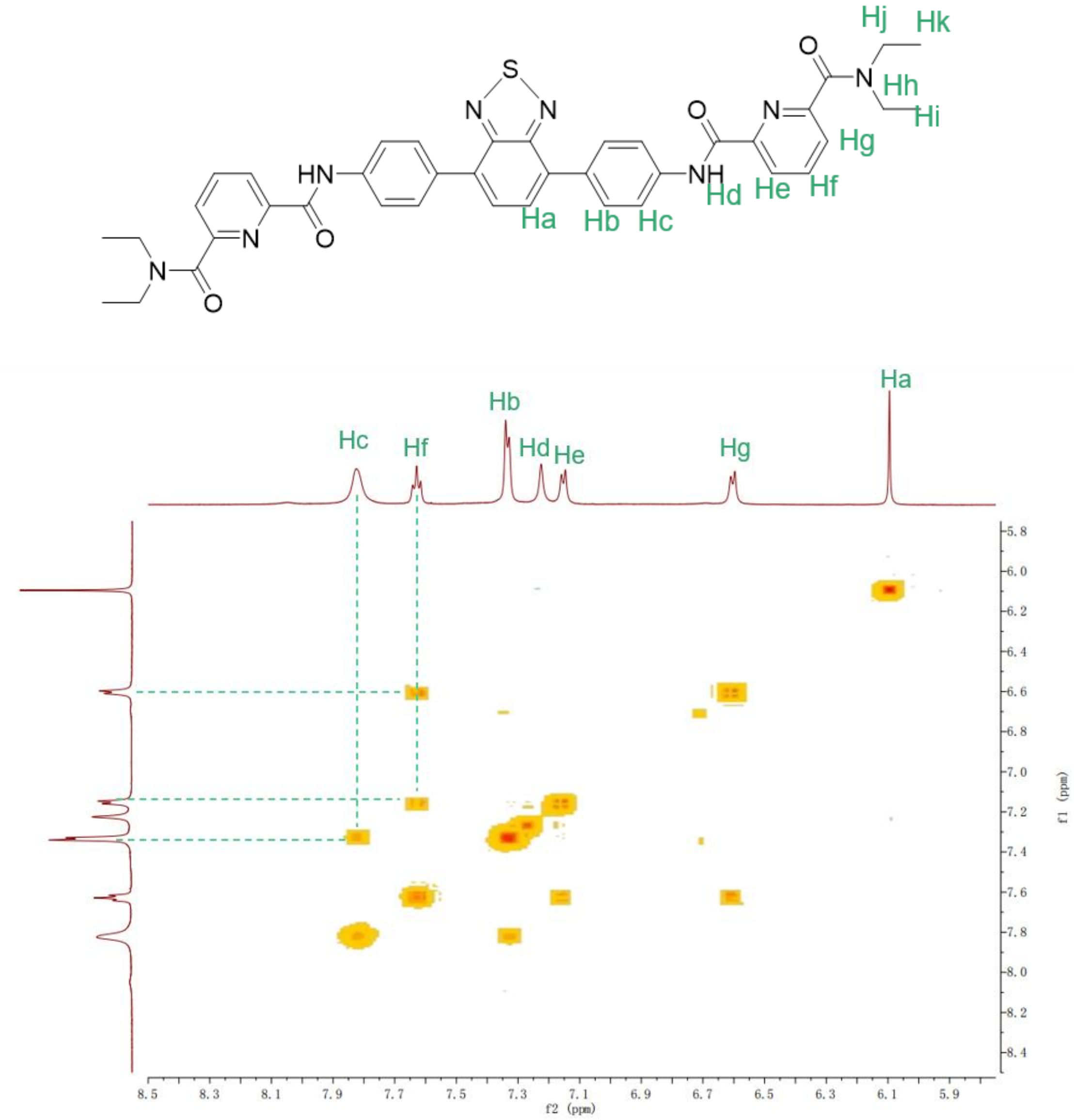

Figure S11. Aromatic region magnification of the COSY (CD_3CN) spectrum of **C**A.

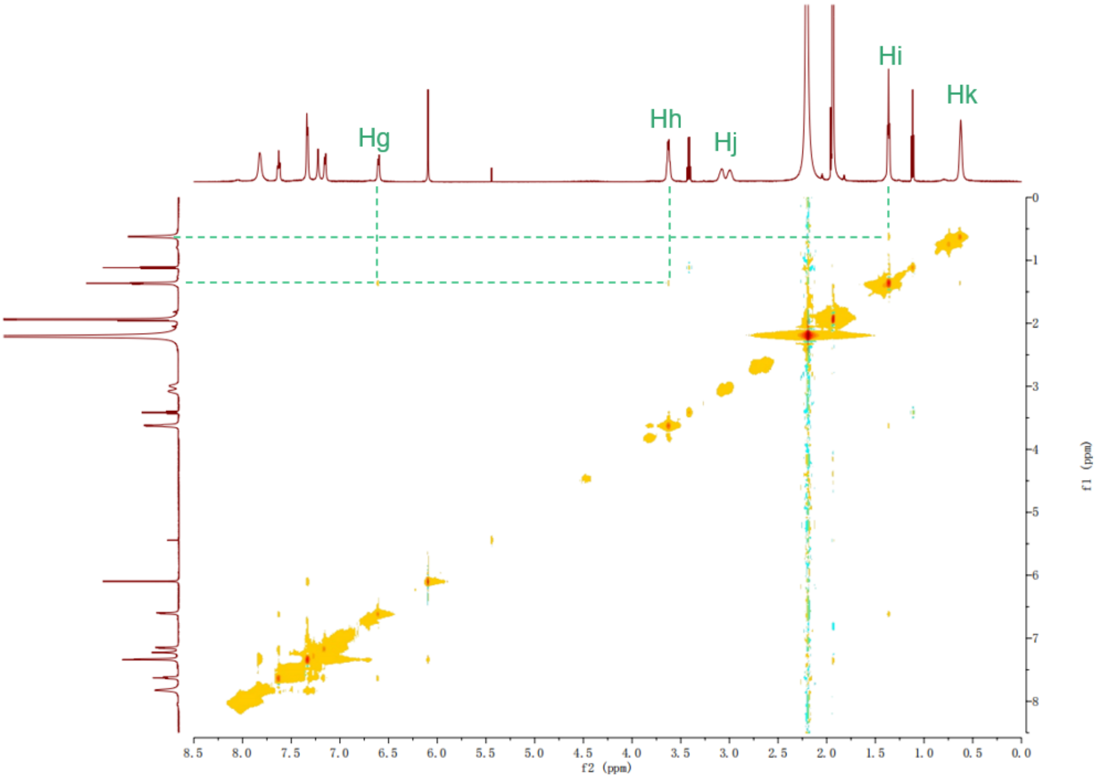

Figure S12. Aromatic region of the NOESY (CD_3CN) spectrum of **C**A.

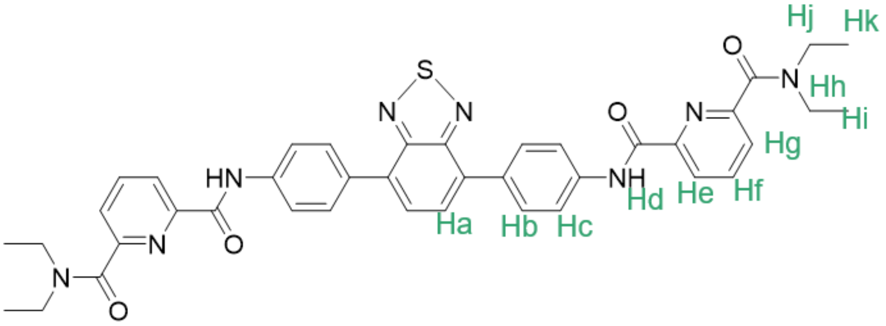

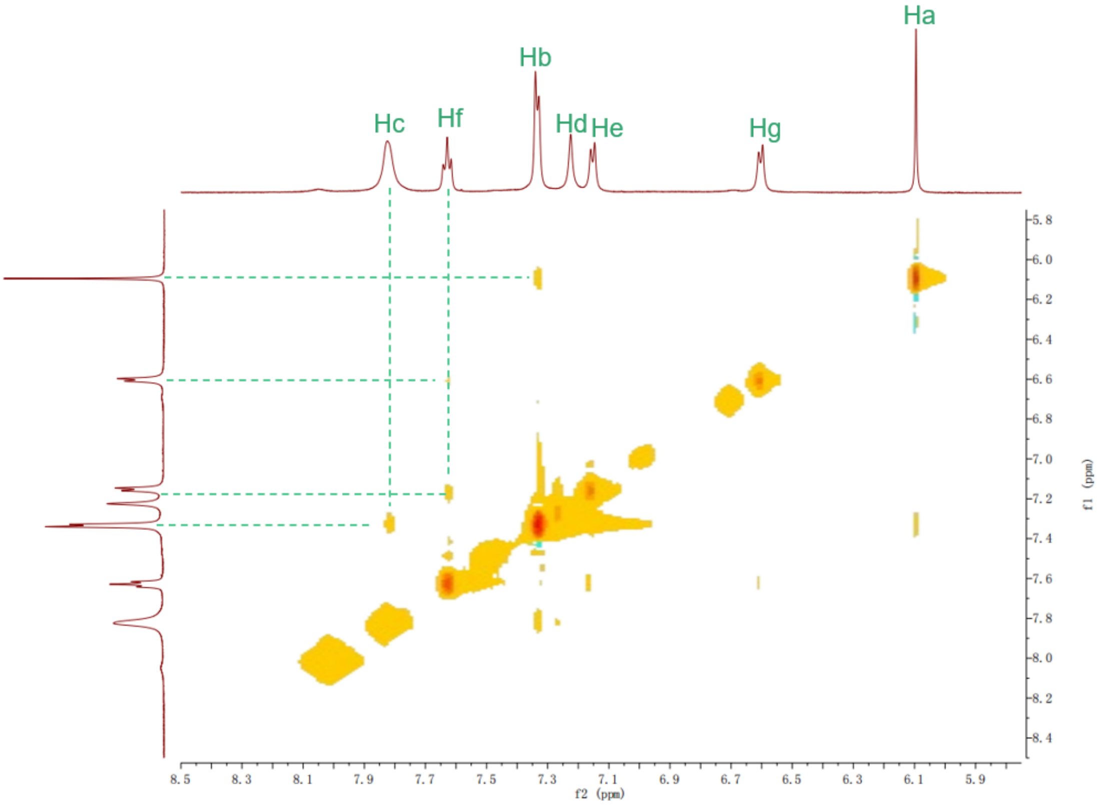

Figure S13. Aromatic region magnification of the NOESY (CD_3CN) spectrum of **C**$_A$.

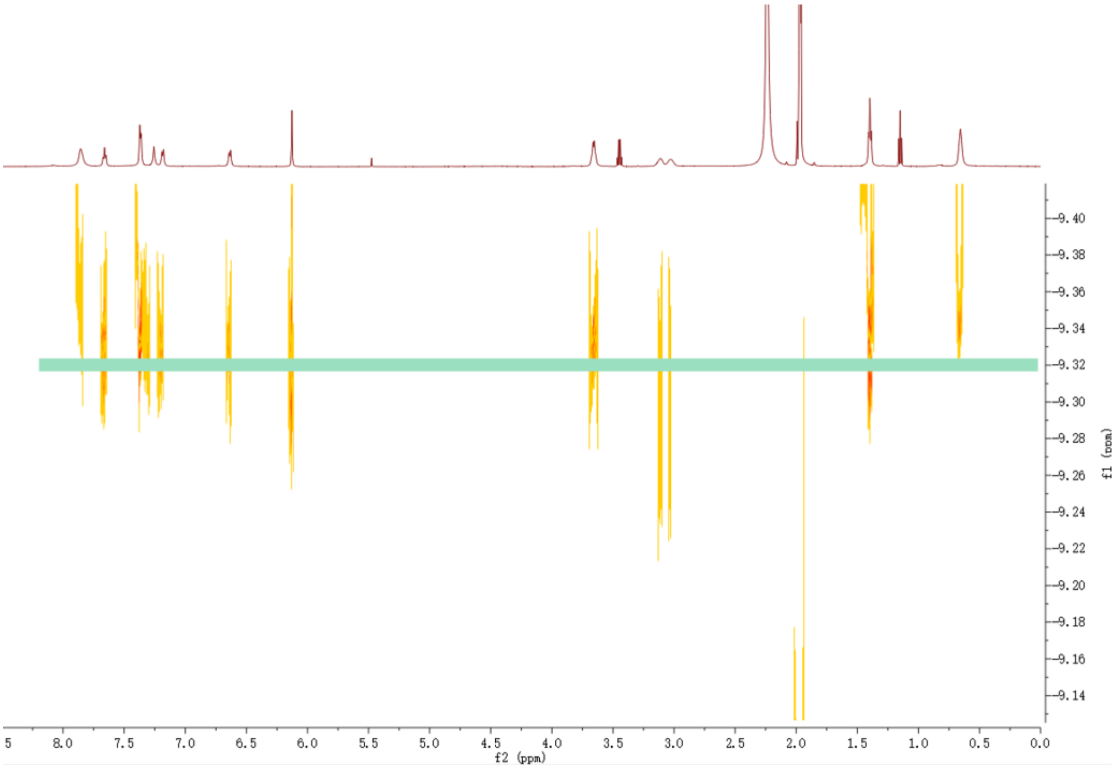

Figure S14. Aromatic region of the DOSY (CD_3CN) spectrum of **C**$_A$.

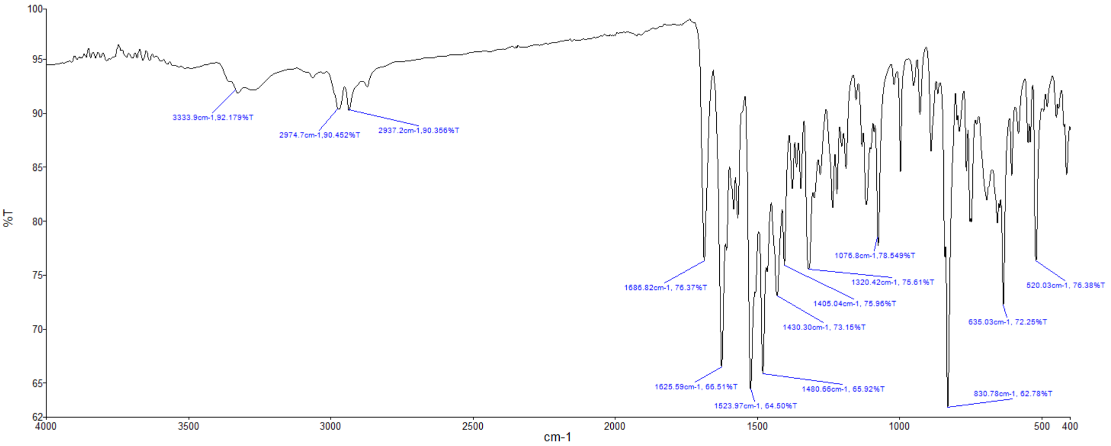

Figure S15. IR spectrum of **L-B**.

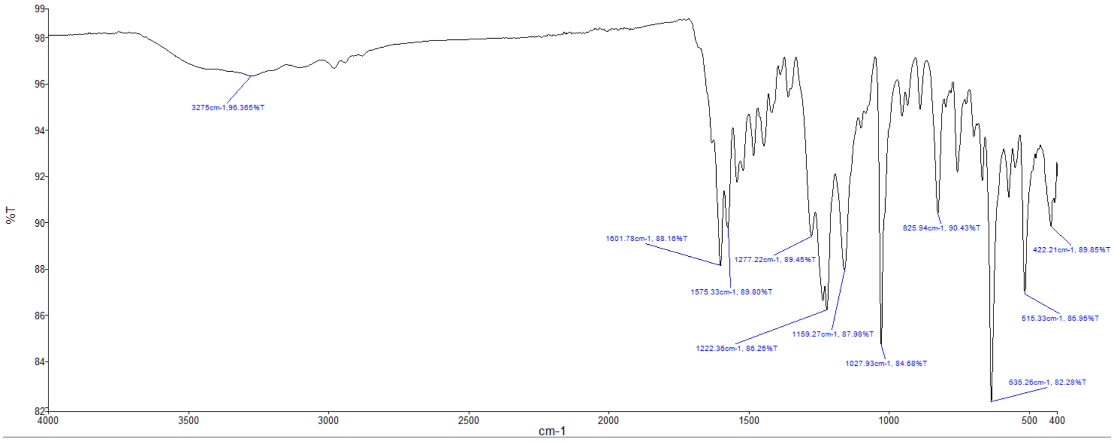

Figure S16. IR spectrum of **C**A.

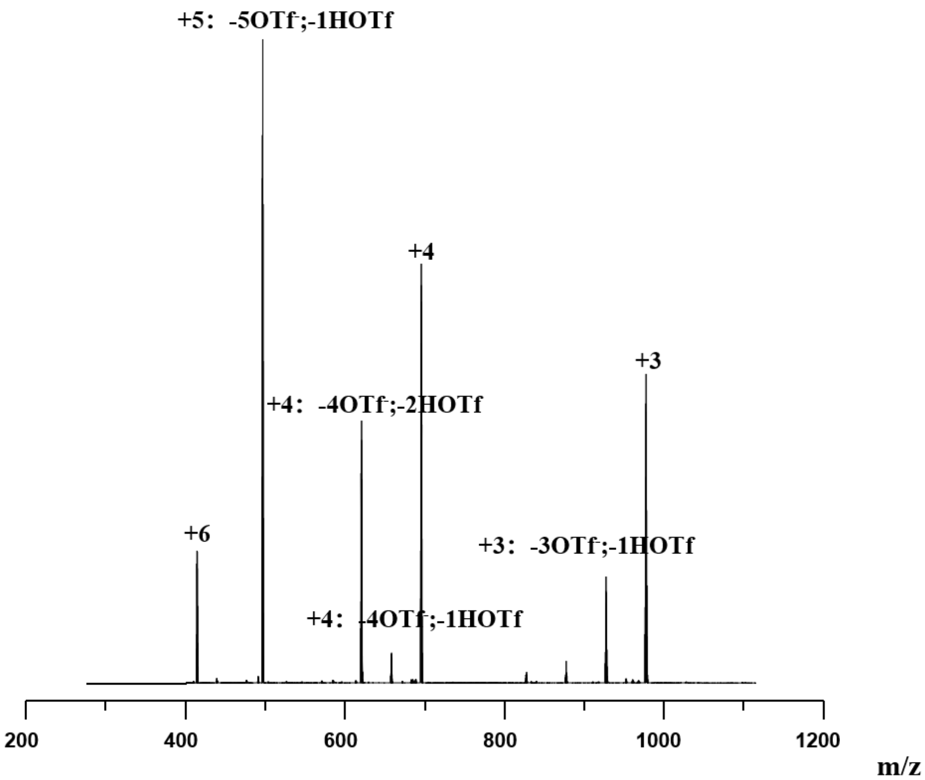

Figure S17. ESI-MS spectrum of **Ca**.

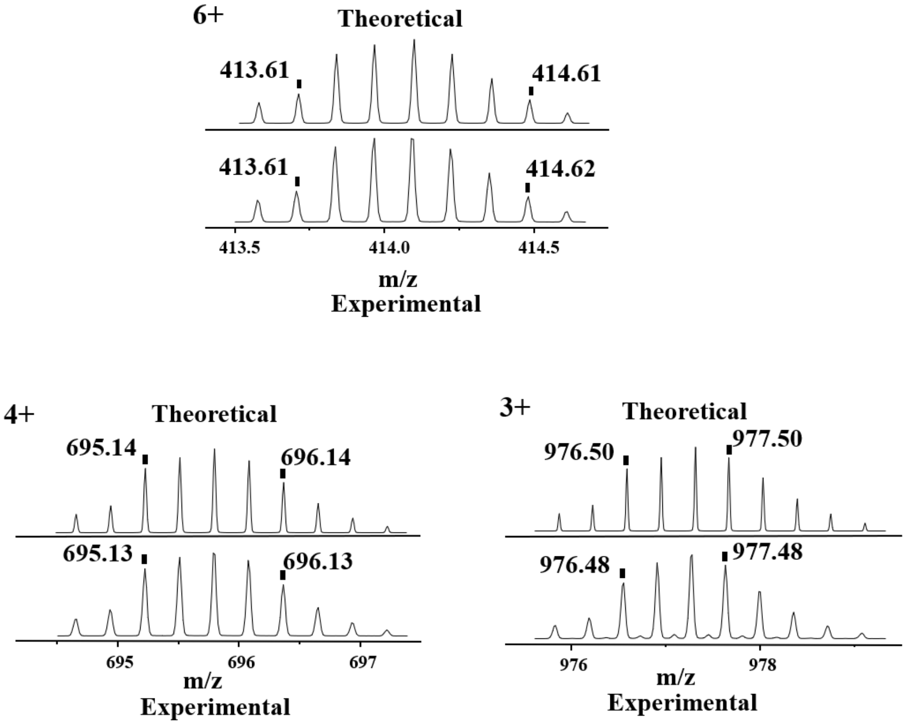

Figure S18. Calculated (top) and Measured (bottom) isotope patterns for different charge states observed from **Ca** (OTf⁻ as counterion).

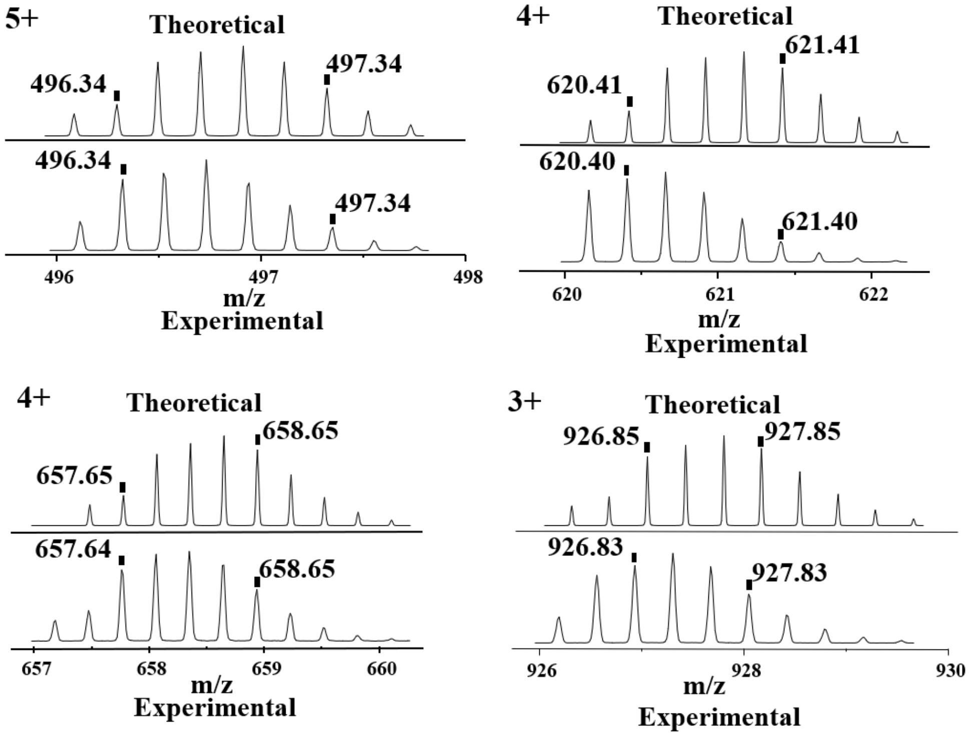

Figure S19. Calculated (top) and Measured (bottom) isotope patterns for different charge states observed from **C**$_{\mathbf{A}}$ (OTf^- and HOTf as counterion).

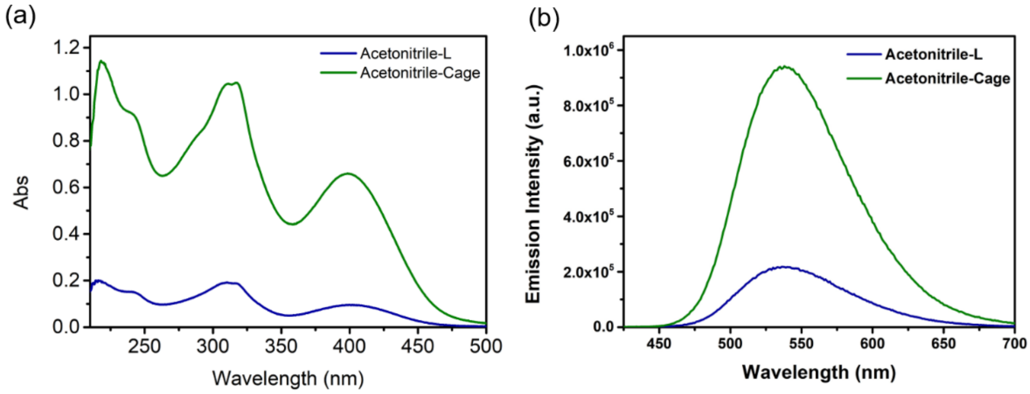

Figure S20. Optical properties of **C**$_{\mathbf{A}}$ and **L-B** in acetonitrile (c=50 ug/mL). (a) UV–Vis absorption. (b) PL emission spectra (λex = 400 nm).

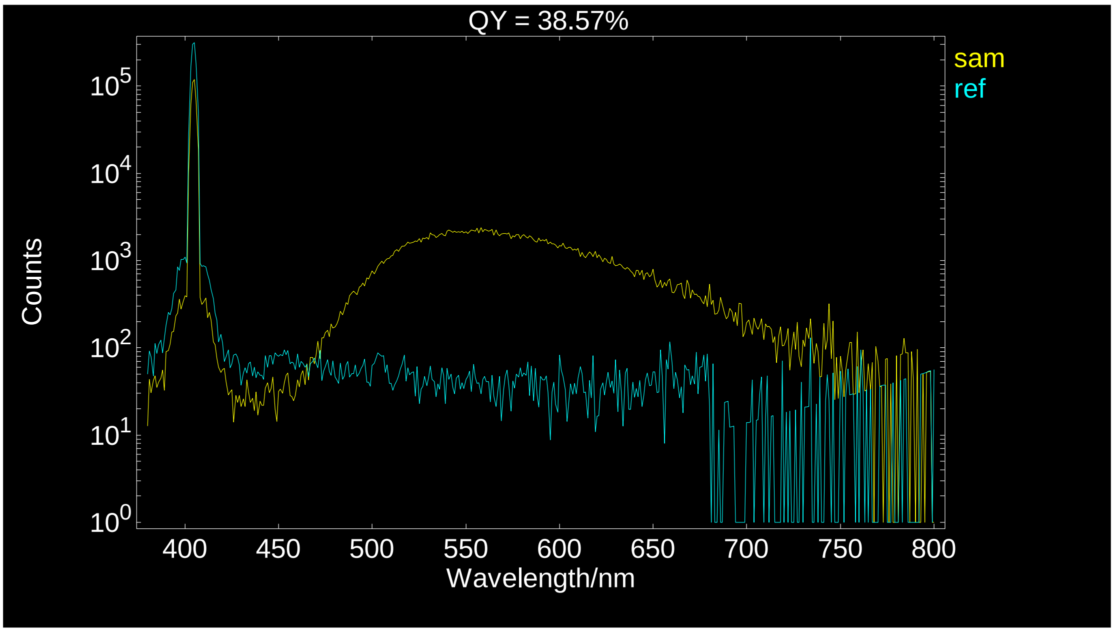

Figure S21. the absolute PL quantum yield of **C$_A$** in acetonitrile (c=50 ug/mL).

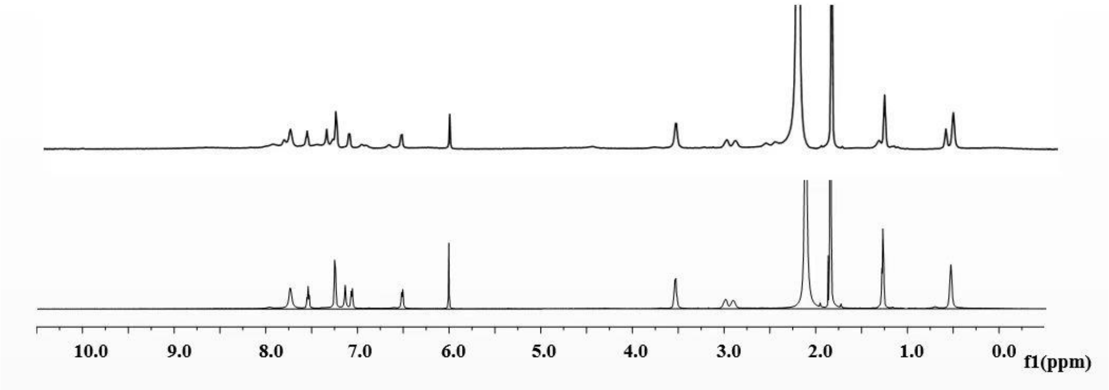

Figure S22. ^{1}H NMR spectra (600 MHz, CD_3CN, 298 K) of **C$_A$** + DMEM. (up), and **C$_A$**. (down).
